# *Allium sativum*: A potential natural compound for NAFLD prevention and treatment

**DOI:** 10.3389/fnut.2023.1059106

**Published:** 2023-02-02

**Authors:** Parham Mardi, Reza Kargar, Ramina Fazeli, Mostafa Qorbani

**Affiliations:** ^1^Non-Communicable Diseases Research Center, Alborz University of Medical Sciences, Karaj, Iran; ^2^Student Research Committee, Alborz University of Medical Sciences, Karaj, Iran; ^3^Chronic Diseases Research Center, Endocrinology and Metabolism Population Sciences Institute, Tehran University of Medical Sciences, Tehran, Iran

**Keywords:** *A. sativum*, NAFLD, fatty liver, NASH, garlic (*A. sativum*)

## Abstract

**Introduction:**

Non-alcoholic fatty liver disease (NAFLD) results from an excessive accumulation of fat particles that causes liver inflammation, which ultimately causes liver damage. There is still considerable uncertainty about the effects of any nutritional supplements compared to no additional intervention. This review aimed to evaluate the efficacy of *Allium sativum (A. sativum)*, known as garlic, in preventing and treating NAFLD.

**Methods:**

A systematic search based on a search strategy consisting of two components of “NAFLD” and “*Allium sativum*” in databases including PubMed, Web of Science (WoS), and SCOPUS was conducted on papers evaluating the effects of *A. sativum* on NAFLD treatment and prevention. We obtained studies from inception until 20 September 2022, followed by study selection and data extraction based on our eligibility criteria. Consequently, qualitative and quantitative synthesis was conducted.

**Results:**

Our qualitative analysis reveals that *A. sativum* consumption is linked to the prevention of NAFLD, especially in males, although qualitative data in this study regarding the therapeutic properties of NAFLD was controversial. Our meta-analysis showed that NAFLD patients treated with *A. sativum* have significantly declined aminotransferase levels. That is to say, our meta-analysis revealed a lower alanine transaminase (ALT) (SMD = −0.580, 95%CI = −0.822 to −0.338), and aspartate transaminase (AST(SMD = −0.526, 95%CI = −0.767 to −0.284) in NAFLD patients treated with *A. sativum* compared to the placebo group. Also, pooling data from case-control studies showed that A. sativum consumption decreases the odds of being diagnosed with NAFLD by 46% (OR = 0.538, 95%CI = 0.451–0.625).

**Conclusion:**

*A. sativum* consumption is not merely associated with NAFLD prevention but also results in a considerable decline in blood aminotransferase levels in patients diagnosed with NAFLD. To put it simply, *A. sativum* is linked to a decline in AST and ALT, which are considered reliable biomarkers of NAFLD response to treatment. Nevertheless, *A. sativum* is insufficient to improve NAFLD independent of other dietary amendments and lifestyle modifications.

## 1. Introduction

Non-alcoholic fatty liver disease (NAFLD) is an excessive accumulation of fat particles that causes liver inflammation, which ultimately causes liver damage (1). NAFLD has become increasingly common in parallel with the increasing prevalence of cardiometabolic risk factors such as obesity, dyslipidemia, type 2 diabetes, and other components of metabolic syndrome (2). Although this condition is mainly diagnosed by ultrasonography, which sometimes is required to be verified by biopsy, guidelines strongly recommend physicians measure aminotransferase enzymes levels, including alanine transaminase (ALT) and aspartate transaminase (AST) each 3-6 months to evaluate their patients’ response to treatment (3–5).

It should be noted that not merely NAFLD as a metabolic disturbance independently increases the risk for cardiovascular morbidity and mortality (6–8), but also its progression can consequently lead to hepatocellular carcinoma (HCC), cirrhosis, and liver fibrosis (9).

Although NAFLD can cause severe hepatic sequels, its pathogenesis mainly involves metabolic disturbances. Its progression is closely associated with dyslipidemia and type 2 diabetes. In other words, previous studies demonstrated that insulin resistance increased levels of triglyceride (TG), and decreased levels of high-density lipoprotein cholesterol (HDL-C) are linked to NAFLD development (10).

Based on its development mechanism, the primary method of NAFLD treatment is lifestyle modification, which includes considerable weight loss, nutritional interventions, frequent exercise, and calorie restriction (11, 12). Moreover, some studies proposed anti-diabetic agents such as Pioglitazone and sodium-glucose cotransporter-2 (SGLT2) inhibitors as potential agents to manage NAFLD patients (13, 14). In contrast, some other studies supported the theory that antioxidant therapy using vitamin E supplementation exerts therapeutic effects in NAFLD patients (15, 16). Nevertheless, no drug has received the necessary approval from FDA (Food and Drug Administration) for NAFLD treatment (9), and efforts to discover therapeutic agents are ongoing. Beside clinical recommendations regarding prescribing pioglitazone and vitamin E in NAFLD patients, drugs such as Dapagliflozin, Aramchol, Resmetirom, Semaglutide, and Lanifibranor have shown promising effects and have entered phase III clinical trials (17).

Meantime, to control NAFLD, many traditional herbal formulas with antioxidant effects are getting studied (18–21). Researchers are also evaluating the impact of herbal agents that have shown beneficial effects in metabolic disturbances to treat NAFLD (20).

*Allium sativum* (*A. sativum*), known as garlic, has been shown to have well-established therapeutic effects on metabolic disturbances such as dyslipidemia and diabetes (22, 23), as well as notable antioxidant activity (24). In other words, previous studies demonstrated that consuming *A. sativum* is attributed to an enhanced lipid profile, altered glucose metabolism, and lower blood pressure (25–27). Moreover, *A. sativum* has shown significant antioxidant activity, which may decrease inflammation in the liver and consequently decrease NAFLD incidence (28). Nonetheless, there is a controversy over the effects of *A. sativum* on NAFLD patients in clinical practice (29–33). This systematic review aims to evaluate the impact of *A. sativum* in the prevention and treatment of NAFLD.

## 2. Materials and methods

This systematic review study was based on the Preferred Reporting Items for Systematic Reviews and Meta-Analyses (PRISMA) statement (34).

### 2.1. Study questions

• Does treatment with *A. sativum* improve the follow-up results (including ALT and AST levels) in patients previously diagnosed with NAFLD?
• Is consuming more *A. sativum* decrease the odds of being diagnosed with NAFLD?

### 2.2. Information sources and search strategy

In the current study, two researchers (MQ and RK) independently conducted the systematic search from inception until September 20, 2022, through PubMed, Scopus, and Web of Science (WoS) databases. The study questions were considered to design the search strategy based on two roots of “NAFLD” and “Allium sativum.” Conflicts were solved by another researcher (PM). Supplementary Table 1 summarizes the search strategy.

### 2.3. Study selection

We performed the study selection process using EndNote reference management software (EndNote 20.1). After including papers based on the search strategy, we initially removed the duplicated papers. Afterward, the titles and abstracts of the documents were evaluated concerning the eligibility criteria, followed by the screening of the full texts of the documents. Two authors (PM and MQ) conducted the study selection.

### 2.4. Eligibility criteria

The following criteria for screening the papers;

• Records should be a clinical trial, a cohort, or a case-control study that includes patients’ *A. sativum* consumption level and‘ their prescribed dosage.
• Records should include data on patients’ NAFLD profiles, including diagnostic or follow-up modalities’ results comprising ultrasonographic studies, histological studies, or aminotransferase enzymes level.
• Records should demonstrate a link between *A. sativum* and patients’ NAFLD-related indices.
• Records can be published in any language. No language restriction was applied.

### 2.5. Data collection process and data items

We designed data extraction forms that include author name, year, provenance, study design, sample size, participants’ age and gender, exposure (*A. sativum*) related indices, outcome (NAFLD-related) indices, the measure of association, confounders, and study findings. Two researchers have filled out extraction forms independently (RK And MQ). Conflicts were solved by another researcher (PM).

### 2.6. Quality assessment (QA)

The quality of cohort and case-control studies was assessed using the Newcastle-Ottawa scale (NOS) statement (35). Moreover, the QA of clinical trials was carried out using the CONsolidated Standards of Reporting Trials (CONSORT) guideline (36). Two researchers independently performed the QA based on the guidelines (RK and MQ).

### 2.7. Data synthesis

Results are presented as standard mean difference (SMD) and their 95% confidence interval (95% CI). STATA version 14 (StataCorp, College Station, TX, USA) software was used to conduct the meta-analysis. We performed a meta-analysis when two or more than two studies report the same measure that shows the association between *A. sativum* and patients’ NAFLD-related indices.

Standard mean differences and their 95%CI pooled estimate were measured using the fixed model on the data extracted from the included clinical trials. In contrast, for case-control studies, a random model meta-analysis was conducted.

The Chi-square-based *Q* test and *I*^2^ statistic were used to evaluate heterogeneity and, consequently, identified the model used for analysis. Lack of heterogeneity was defined when the p-value was more than 0.10. Regression and Galbraith analyses were used to identify the source of heterogeneity in studies. Publication bias assessment was carried out using Egger’s regression asymmetry test. Lack of publication bias was defined when the *p*-value was more than 0.10.

Although it is challenging to interpret and might lead to crucial methodological bias, to pool all studies showing the overall link between *A. sativum* and NAFLD, we transformed SMDs into ORs based on Cochran handbook 5-1 (part 2: general methods for Cochrane reviews) (37). This process was followed by pooling ORs.

## 3. Results

### 3.1. Systematic review results

**FIGURE 1:**
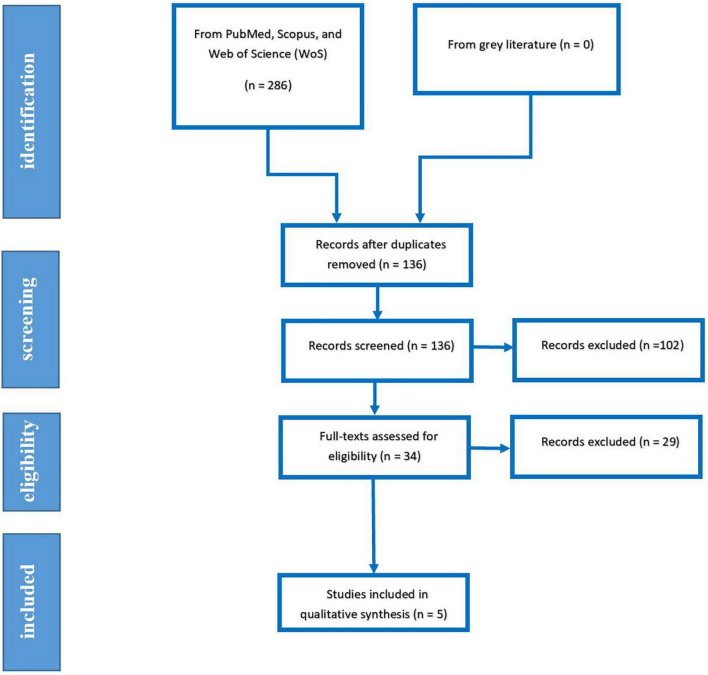
PRISMA flow diagram.

Our systematic search yielded 286 papers, which was eliminated to 136 papers after removing duplicated papers. After evaluating the papers’ title, abstract, and full text, 5 Records, including three RCTs and two case controls, were eventually included in qualitative and quantitative analysis. The PRISMA flow diagram of systematic search is demonstrated in Figure 1.

### 3.2. Characteristics of included studies

As previously mentioned, two case-control (29, 33) and three randomized controlled trial studies (30–32) were included in the meta-analysis separately. All studies were conducted in Asia, and three out of five included studies were conducted in Iran. Table 1 provides the characteristics of included studies. A total of 25380 participants (11917 males) were assessed in included records.

**TABLE 1:** Characteristics of studies that were included in the qualitative analysis.

Author	Year	Provenance	Study design	Sample size	Age Mean ± SD	Gender male (%)	Quality score
Emamat et al. (29)	2020	Iran	Case-control	999	Case: 42.3 ± 11.9 Control: 43.5 ± 14.5	433 (43.3)	7/9*
Kim et al. (30)	2017	Republic of Korea	Randomized-Double blinded Clinical Trial	75	Treatment: 54.5 ± 12.2 Control: 54.2 ± 9.3	55 (73.3)	31/37**
Sangouri et al. (31)	2020	Iran	Randomized-Double blinded Clinical Trial	90	Treatment: 45.2 ± 12.4 Control: 44.2 ± 11.1	57 (63.3)	28/37**
Soleimani et al. (32)	2020	Iran	Randomized-Double blinded Clinical Trial	110	Treatment: 45.6 ± 11.3 Control: 42.9 ± 12.21	46 (41.8)	30/37**
Zhang et al. (33)	2019	China	Case-control	24,106	Male: 41.0 ± 12.2 Female: 40.3 ± 11.6	11,326 (47.0)	7/9*

*Quality assessment was conducted based on Newcastle – Ottawa Quality Assessment Scale (NOS) checklist for case-control studies.

**Quality assessment was conducted based on the CONSORT checklist.

### 3.3. Qualitative analysis

The qualitative analysis of this review comprises two sections; Emamat et al. revealed that patients consuming less *A. sativum* are at higher odds of being diagnosed with NAFLD based on the ultrasonographic method (29). In contrast, Zhang et al. study revealed that consuming raw garlic more than seven times a week only decreases the odds of NAFLD by 29 (OR = 0.71, 95%CI: 0.55–0.90) percent in males while leading to insignificant findings in females. Notably, their findings were insignificant in females (OR = 1.17, 95%CI: 0.86–1.58) (33). The second section assessed included trials. All randomized clinical trials revealed decreasing properties of *A. sativum* for ALT level (30–32), while two (31, 32) out of three included clinical trials demonstrated a significant decline in AST following treatment with *A. sativum.* It should be considered that data regarding GGT were not parallel Table 2.

**TABLE 2:** Qualitative analysis showing the association of garlic and NASH related indices.

Author, date (journal name)	Garlic related	Liver index	Measure of association	Test results		Confounders
Emamat et al. (29)	Consumption of garlic (g/day)	NASH*	Mann–Whitney test	NASH group garlic consumption Median(IRQ) N = 198	3.76 (0.78–12.27)	Age, gender, BMI, energy intake and physical activity
				Normal group garlic consumption Median(IRQ) N = 803	9.29 (4.43–16.06)	
				*p*-Value	<0.001	
			OR (T3 vs. T1)^1^	OR (95%CI)	0.36 (0.22–0.56)	
			OR (T2 vs. T1)^2^	OR (95%CI)	0.47 (0.30–0.71)	
Kim et al. (30)	Fermented garlic extracts (FGE, dose = 1.5 g/day) N = 36	GGT	Mean and 95%CI of Enzymatic activities in circulating blood	Baseline	90.9 (79.0–105.6)	Not adjusted
				12-week visit	79.8 (65.3–97.5)	
		AST		Baseline	30.5 (26.0–35.8)	
				12-week visit	27.6 (23.5–32.4)	
		ALT		Baseline	35.5 (29.3–42.5)	
				12-week visit	32.1 (26.3–38.8)#	
		AST/ALT		Baseline	0.86 (0.76–0.99)	
				12-week visit	0.86 (0.76–0.98)	
	Placebo N = 39	GGT		Baseline	86.4 (75.1–100.4)	
				12-week visit	95.5 (79.0–115.5)	
		AST		Baseline	28.5 (25.0–32.7)	
				12-week visit	30.8 (27.1–34.8)	
		ALT		Baseline	30.2 (25.5–35.5)	
				12-week visit	35.5 (30.5–41.2)#	
		AST/ALT		Baseline	0.94 (0.84–1.06)	
				12-week visit	0.87 (0.76–0.98)	
	Group × time interaction	GGT	Group × time Interaction, F	3.98#		
		AST		2.14		
		ALT		3.28#		
		AST/ALT		1.17		
Sangouni et al. (31)	Garlic Tablets dose = 6 mg of allicin/day N = 45	ALT	Mean ± SD of Enzymatic activities in circulating blood	Baseline	30.9 ± 15.7	mean changes of WC and baseline values of parameters
				12-week visit	26.0 ± 13.2	
		AST		Baseline	22.8 ± 11.1	
				12-week visit	20.6 ± 8.6	
		GGT		Baseline	32.0 ± 21.0	
				12-week visit	29.0 ± 16.4	
		ALP		Baseline	203.8 ± 56.9	
				12-week visit	200.3 ± 49.0	
	Placebo N = 43	ALT		Baseline	24.9 ± 15.4	
				12-week visit	28.5 ± 17.6	
		AST		Baseline	21.2 ± 7.3	
				12-week visit	23.1 ± 9.4	
		GGT		Baseline	28.7 ± 12.0	
				12-week visit	29.5 ± 12.3	
		ALP		Baseline	190.9 ± 63.1	
				12-week visit	190.0 ± 56.7	
	Group × time interaction	ALT	Group × time Interaction, P	< 0.001		
		AST		0.01		
		GGT		0.02		
		ALP		0.65		
Soleimani et al. (32)	Garlic Tablets dose = 6 mg of allicin/day N = 55	AST	Mean ± SD of Enzymatic activities in circulating blood	Baseline	48.3 ± 11.6	energy intake and physical activity
				15-week visit	42.2 ± 11.24	
		ALT		Baseline	57.8 ± 13.9	
				15-week visit	47.2 ± 16.1	
	Placebo N = 55	AST		Baseline	45.6 ± 11.2	
				15-week visit	47.39 ± 12.5	
		ALT		Baseline	55.3 ± 15.2	
				15-week visit	55.5 ± 19.4	
	Group × time Interaction	AST	Group × time Interaction, P	<0.001		
		ALT		<0.001		
Zhang et al. (33)	Garlic (≥7t/week vs. <1t/week)^2^	NASH*	OR	OR (95%CI) in males	0.71 (0.55, 0.90)	Age, BMI, educational level, occupation, household income, PA, and total energy intake, and three major dietary patterns (raw garlic and total onions were not included in the calculation) and total onion intake.
	Garlic (4–6t/week vs. <1t/week)^2^				0.66 (0.54, 0.80)	
	Garlic (1–3t/week vs. < 1t/week)^2^				0.81 (0.73, 0.90)	
	Garlic (≥7t/week vs. <1t/week)^2^			OR (95%CI) in females	1.17 (0.86, 1.58)	
	garlic (4–6t/week vs. <1t/week)^2^				1.17 (0.88, 1.55)	
	Garlic (1–3t/week vs. <1t/week)^2^				1.12 (0.96, 1.30)	

*NASH was diagnosed based on ultrasonography examination.

^1^T1 = 0.19–2.52 g/day of garlic, ^2^total consumption over the previous month (times per week). T3 = 15.21–32.55 g/day of garlic. IRQ, interquartile range; OR, odds ratio; CI, confidence interval.

### 3.4. Quantitative analysis

#### 3.4.1. Clinical trials (therapeutic properties)

**FIGURE 2:**
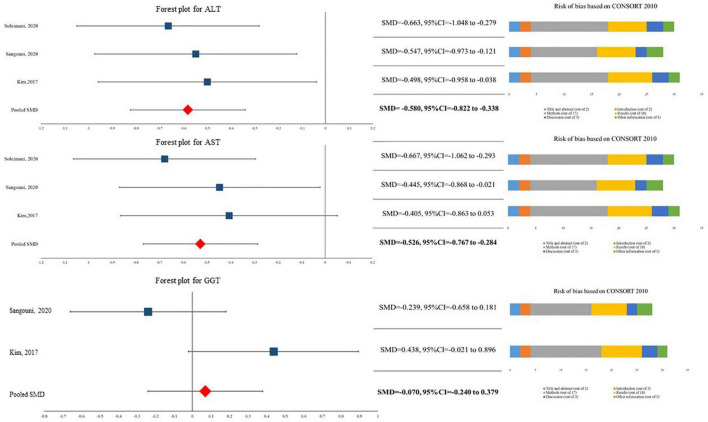
Findings of quantitative synthesis pooling SMDs.

The current meta-analysis showed that patients in *A. sativum* have significantly 9.479 U/L (95%CI for MD = −13.350 U/L to −5.608 U/L) lower levels of ALT compared to their peers in the placebo group (SMD = −0.580, 95%CI = −0.822 to −0.338). Similarly, our data indicated that *A. sativum* results in significantly lower levels of AST (SMD = −0.526, 95%CI = −0.767 to −0.284). It should be noted that this quantitative synthesis did not demonstrate a significant difference between patients in the *A. sativum* group and placebo group in terms of GGT level (SMD = −0.070, 95%CI = −0.240 to −0.379) (Figure 2). As *I*^2^ was reported at 0.00% in all three analyses, we assumed heterogeneity was not considerable. Likewise, no evidence of publication bias was reported in Eggers’s test.

#### 3.4.2. Case-controls (preventive properties)

**FIGURE 3:**
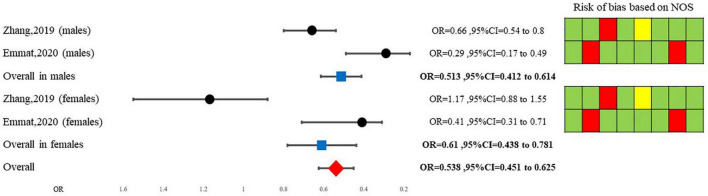
Findings of quantitative synthesis pooling ORs.

Our random model meta-analysis showed that overall, *A. sativum* consumption decreases the odds of being diagnosed with NAFLD by 46% (OR = 0.538, 95%CI = 0.451–0.625). Due to high heterogeneity among studies, a Galbraith and a meta-regression analysis were conducted. Initially, Galbraith’s synthesis revealed that Zhang et al. study for females is an outlier study. After its exclusion, our fixed model decreased OR to 0.492 (95%CI = 0.402–0.582). Furthermore, meta-regression analysis showed that while the gender of participants may be a source of heterogeneity, other variables such as age, NAFLD diagnostic method, body mass index, diabetes diagnosis, or lipid profile may not be linked to heterogeneity among studies. Sub-group analysis based on gender is illustrated in Figure 3.

#### 3.4.3. Overall

Current analysis showed a significant link between *A. sativum* and NAFLD (pooled OR = 0.495, 95%CI: 0.418–0.572).

## 4. Discussion

In this systematic review and meta-analysis, we analyzed studies that examined the effect of *A. sativum* consumption on NAFLD. Our systematic review and meta-analysis show that *A. sativum* extract prescription for NAFLD patients significantly reduces ALT and AST levels. In addition, this study shows that consuming more *A. sativum* is correlated with a lower risk of being diagnosed with NAFLD.

Although there was a controversy over the clinical effect of *A. sativum* on NAFLD patients, previous studies revealed possible biological justifications. Various medicinal properties have been reported for *A. sativum*, including lowering cholesterol and blood pressure, anti-platelet effects, antimicrobial activities, and preventing cancer (38–41). The hepatoprotective effects of *A. sativum* are due to its water-soluble and fat-soluble compounds. Water-soluble compounds comprise of g-glutamyl S-allyl-cysteine (SAC) group, including SAC and S- allyl-mercapto cysteine (SAMC) and lipid-soluble compounds include allyl sulfur compounds such as diallyl disulfide (DADS) and diallyl trisulfide (DATS) (42, 43). It is demonstrated that SAC induces the AMPK pathway by activating calcium/calmodulin-dependent kinase (CaMKK) and silent information regulator T1 (SIRT1), inhibiting hepatic lipotoxicity and lipogenesis.

Moreover, it can reduce cell death caused by lipid accumulation by suppressing free fatty acid (FFA)-induced reactive oxygen species production and caspase activation (44). The antifibrotic, anti-inflammatory and antioxidant effects of SAMC have been observed in a study by Xiao et al. SAMC alleviates fibrosis by reducing Transforming Growth Factor-β1 and α-Smooth Muscle Actin and inhibiting the activation of hepatic Kupffer cells and hepatic stellate cells (HSCs). SAMC exerts anti-inflammatory effects by lowering the nuclear transcription factors NF-κB and AP-1. SAMC also protects the liver from hepatic oxidative stress by inhibiting cytochrome P450 2E1 and elevating antioxidant enzymes glutathione peroxidase and catalase (45). Furthermore, upregulating the expression of lipolytic genes peroxisome proliferator-activated receptor α and carnitine palmitoyltransferase-1 and down-regulating the expression of lipogenic gene sterol regulatory element-binding protein-1c by DATS reported, which can prevent the progression of NAFLD (46). A recent study by Zhang et al. (43) revealed that DATS could mitigate the profibrogenic process and oxidative stress during NAFLD mediated by Hydrogen Sulfide production in HSCs. To sum up, the composition of *A. sativum* has played a crucial role in preventing and treating NAFLD.

To illustrate the effectiveness of *A. sativum* in preventing and treating NAFLD, a comparison of its efficacy with routine and herbal treatments commonly used for NAFLD is vital. Currently, there are no FDA-approved therapies for the disease. However, lifestyle modifications, including dietary changes, weight loss, and increased physical activity, are generally recommended for these patients. Besides, vitamin E and pioglitazone are usually employed to treat patients.

The efficacy of weight loss in improving NAFLD has been shown in several studies. That is to say, in 97% of people who have lost more than 10% of their total body weight, the resolution of the disease occurs. It should be noted that only 10–20% of people can lose more than 10% of their weight in 1–2 years (47). In other words, the success rate of this method is significantly reduced due to its low participation rate. In this respect, treatment with *A. sativum* may be superior because it is more likely be better tolerated.

On the other hand, vitamin E, which has antioxidant properties, is one of the therapies suggested for fatty liver. Numerous studies have reported that vitamin E, similar to *A. sativum*, can modulate the aminotransferase level. Vitamin E also improves histological abnormalities; However, no significant effect of this vitamin on liver fibrosis has been observed (48). It should be noted that a cohort study revealed that a six-month course of vitamin E therapy, in contrast to *A. sativum* consumption, did not alter patients’ metabolic profile, body weight, and lipid and glucose levels (49).

In addition to routine treatments, several natural products have been suggested to control liver steatosis. Utilizing natural products as an elective approach to treating NAFLD has drawn developing attention among doctors. Consequently, researchers are using various pipelines to determine the effects of natural products to control NAFLD. Many of these products are being assessed in both animal and human subjects (17, 50).

Similar to the current study, Goodarzi et al. (51) demonstrated a significant decline in the serum concentrations of ALT and AST following turmeric/curcumin supplementation. Silymarin also significantly reduces levels of ALT and AST. However, it does not affect BMI (52). Although resveratrol administration in animal studies has reduced inflammation and liver steatosis (53), there is a controversy over its effects on human participants. While some studies have found Resveratrol to be effective in reducing liver enzymes as well as metabolic parameters (54), others have shown no significant effect on either aminotransferases or metabolic factors (55, 56).

Conversely, there are no such controversies over the effects of *A. sativum* on NAFLD. A recent study showed that baseline health status is a significant confounder in the outcome of green tea on liver enzyme and bilirubin levels. While green tea decreases liver enzymes level in NAFLD patients, it leads to a notable increase in liver enzymes in healthy individuals (57). Therefore, green tea, unlike *A. sativum*, could not be a suitable option for prevention.

In this systematic review, we included not merely the frequency of patients diagnosed with NAFLD but also their aminotransferase level (58). Although liver biopsy is the gold standard for diagnosing NAFLD, AST and ALT levels are suggested for follow-up of patients previously diagnosed (59). It is shown that the proportion of NAFLD patients with normal ALT is significantly lower than individuals with elevated ALT levels. However, the aminotransferase levels could not predict advanced fibrosis in NAFLD patients (60, 61). Nevertheless, the need for further studies to determine the effect of *A. sativum* on the fatty liver by evaluating more specific outcomes, such as biopsies, to confirm the current results is undeniable.

This study had several limitations; The number of included studies and, consequently, the overall sample size were limited in the current study. There was also a difference in the design of included studies. It should be considered that the duration of treatments in RCTs varied from 12 to 15 weeks. More importantly, different studies used different doses of *A. sativum*, which makes it challenging to mirror the findings of the current review to clinical practice.

Not merely consuming more *A. sativum* is attributed to lower odds of being diagnosed with NAFLD, but also consuming garlic significantly decreases AST and ALT levels in patients previously diagnosed with NAFLD. In other words, along with other dietary amendments and lifestyle modifications, *A. sativum* can be used in two clinical perspectives and approaches. First, it can be prescribed for people with NAFLD to slow down the progression of the disease. Second, taking *A. sativum* as prophylaxis in high-risk participants decreases the odds of being diagnosed with NAFLD.

## Data availability statement

The original contributions presented in this study are included in the article/Supplementary material, further inquiries can be directed to the corresponding author.

## Author contributions

PM participated in drafting the manuscript, participated in the systematic search, and conducted the analysis. RK participated in the systematic search. RF participated in drafting the manuscript. MQ participated in a systematic search, revised the manuscript, and supervised the project. All authors contributed to the article and approved the submitted version.
