# Supplementary material for: Allium sativum: A potential natural compound for NAFLD prevention and treatment
Source: Front Nutr. 2023 Feb 2;10:1059106. doi: 10.3389/fnut.2023.1059106 (PMC9931905; doi:10.3389/fnut.2023.1059106)
Supplement: Supplementary file 1 [file Table_1.DOCX]

**Supplementary table 1**

| 1. | “Non alcoholic Fatty Liver Disease” OR “NAFLD” OR “Nonalcoholic Fatty Liver Disease” OR “Nonalcoholic Steatohepatitis” OR “Nonalcoholic Fatty Liver” |
| --- | --- |
| 2. | “garlic” OR “allium sativum” OR “A. sativum” OR “A sativum” |
| 3. | #1 AND #2 |
| Filters | No language restriction; from inception, until September 20, 2022; |
